# The performance of a modified EWMA control chart for monitoring autocorrelated PM2.5 and carbon monoxide air pollution data

**DOI:** 10.7717/peerj.10467

**Published:** 2020-12-15

**Authors:** Yadpirun Supharakonsakun, Yupaporn Areepong, Saowanit Sukparungsee

**Affiliations:** Department of Applied Statistics, King Mongkut’s University of Technology North Bangkok, Bangkok, Thailand

**Keywords:** Autocorrelation, Average run length, Explicit formulas, PM2.5, Carbon monoxide, Air pollutant

## Abstract

PM2.5 (particulate matter less than or equal to 2.5 micron) is found in the air and comprises dust, dirt, soot, smoke, and liquid droplets. PM2.5 and carbon monoxide emissions can have a negative impact on humans and animals throughout the world. In this paper, we present the performance of a modified exponentially weighted moving average (modified EWMA) control chart to detect small changes when the observations are autocorrelated with exponential white noise through the average run length evaluated (ARLs) by explicit formulas. The accuracy of the solution was verified with a numerical integral equation method. The efficacy of the modified EWMA control chart to monitor PM2.5 and carbon monoxide air pollution data and compare its performance with the standard EWMA control chart. The results suggest that the modified EWMA control chart is far superior to the standard one.

## Introduction

One of the world’s greatest health and environmental problems is air pollution, which has steadily increased worldwide due to global industrialization and urbanization (Manisalidis et al., 2020). Air quality monitoring in many countries has shown that the levels of common pollutants have increased from 1980 until now. During 2010–2016, an estimated 55.3% of the global population were exposed to dangerous levels of air pollution (Shaddick et al., 2020). Therefore, a major global health risk to the world’s population is air pollution, with more than 90% of people living in areas that do not meet the 2017 World Health Organization’s (WHO) recommended threshold of air quality levels (Health Effects Institute, 2019). The WHO data also reveals that in 2016, 6.1 million deaths worldwide (around 11% of the total global deaths) are attributable to air pollution.

A widespread air pollutant consisting of a mixture of solid and liquid particles suspended in air is called particulate matter (PM), in which the physical and chemical characteristics depend on the location. The common chemical components of PM include nitrates, sulfates, ammonium, other inorganic, and biological compounds (Huang et al., 2014). PM is used as an indicator that is relevant to health and is classified in terms of the diameter or width of the particles, e.g., PM10 means the diameter is 10 µm or less and PM2.5 is 2.5 µm or less (Saarikoski et al., 2018). PM2.5 can be human-made and naturally occurring. Air pollution from vehicle emissions and traffic congestion produces both PM2.5 and carbon monoxide (CO) pollution, with major causes being diesel engine exhaust fumes and traffic congestion (Pui, Chen & Zuo, 2014; Tian et al., 2015). Emissions from factories and power plants are also major causes of PM2.5 pollution due to burning fossil fuels, especially coal (Hua et al., 2015). Moreover, the burning of agricultural waste, especially in South East Asia, and forest fires are other major causes of PM2.5 pollution (He, Lui & Zhou, 2020; Lee et al., 2018).

The small size of PM2.5 particles makes them particularly deleterious to health by directly cause respiratory and cardiovascular morbidity. Since the lungs are the primary organs affected by PM2.5, exposure can lead to lung injury by perturbations of the lung microbiome and its associated metabolome mechanism (Li et al., 2020), as well as causing respiratory diseases and lung cancer (Xing et al., 2016). However, PM2.5-induced neuroinflammation and metabolic turbulence may be mitigated by the anti-inflammatory and anti-oxidative activities of fisetin (Xu et al., 2020). Long-term exposure to PM pollution in the air causes extrinsic skin aging, (wrinkles and changes in pigmentation). Moreover, atmospheric pollutants also lead to skin diseases such as atopic dermatitis (Liao, Eie & Sun, 2020).

The trend in Central and Southern Asia has been a rise in PM2.5 levels between 2010 and 2016 (Shaddick et al., 2020; Cheong et al., 2019; Johnston et al., 2019). Recently in Thailand, especially in Bangkok and the surrounding area, the population has been exposed to dangerously high PM2.5 pollution levels due to agricultural waste burning, foreign sources, and industry. The levels of these small particles have increased over the past few years (Wimolwattanapun, Hopke & Pongkiatkul, 2011; Oanh et al., 2013; Pinichka et al., 2017; Mahidol University, 2020). Self-care practices should be the first priority to manage this problem, while PM2.5 pollution levels should be kept under control and closely monitored periodically.

Statistical process control is a quality control approach for carrying out statistical methods to monitor and control process change. In particular, the control chart is a tool that is widely used to monitor processes to detect any changes in them, thereby preventing the occurrence of faults. In many ecological and environmental sources of data, the values are not independent over time and often comprise autocorrelated observations (autocorrelation or serial correlation is a measure of the correlation between current variable values and their past ones). Hence, the assumption in traditional control chart methodology that the observations taken from the process are independent and normally distributed does not hold. Thus, traditional control charts such as the Shewhart control chart have drastically reduced efficiency when applied to serially correlated observations. Therefore, many researchers have proposed the cumulative sum (CUSUM) (Page, 1954) and exponentially weighted moving average (EWMA) (Roberts, 1959) control charts as suitable alternatives for when the observations are autocorrelated. The EWMA control chart is recommended when the observations are not normally distributed or are autocorrelated, as has been determined using the average run length (ARL) statistic (Srivastava & Wu, 1993; Wardell, Moskowitz & Plante, 1994; Jiang, Tsui & Woodall, 2000; Carson & Yeh, 2008; Han et al., 2010). The usefulness of control chart techniques has been investigated for air pollution data in a time series. The CUSUM control chart has been employed for identifying changes in the mean air pollution level (Barratt et al., 2007), as well as for identifying important change-points in the time series of air pollutants measured at a busy roadside location in central London (Carslaw, Ropkins & Bell, 2006). The applicability of the CUSUM control chart for detecting changes in air pollutant concentrations in Delhi was investigated by Chelani (2011), who found that they have been significantly increasing over time. Moreover, standard CUSUM and EWMA control charts have been used to detect a change in air pollutant time series data in Kuwait (Al-Rashed, Al-Mutairi & Attar, 2019); the results reveal that both procedures provided early alarms in the detection of changes in air pollutants. Afterward, a newly structure control statistic was proposed by Patel & Divecha (2011) and was also generalized structure of control statistic namely modified EWMA control chart. Several researchers investigated the performance of modified EWMA chart by different situations of non-normal distribution including Aslam et al. (2017), Herdiani, Fandrilla & Nurtiti (2018) and Noiplab & Mayureesawan (2019). The application of modified EWMA procedures was demonstrated using real-life samples by comparing with the existing charts. The results showed that the proposed control chart is efficient in quick detection of the out-of-control process (Khan et al., 2018; Saghir, Ahmad & Aslam, 2019; Saghir et al., 2020; Aslam & Anwar, 2020; Supharakonsakun, Areepong & Sukparungsee, 2020).

According to these prior studies on control chart performance, the ARL is utilized to measure the robustness of the chart. In this paper, a modified EWMA control chart, which was newly developed from the traditional EWMA procedure, is presented for monitoring and detecting small and abrupt changes in autocorrelated data by evaluating the ARL. Its performance was studied comparatively with the standard EWMA chart for detecting changes in PM2.5 and CO gas level.

### Modified EWMA chart

Roberts (1959) first proposed the EWMA control chart that is very effectively at detecting small process changes. This chart’s design parameters are the multiples of widths of the control limit and smoothing parameter. The EWMA statistic is defined as

(1) \begin{eqnarray*}& & {Z}_{t}=(1-\lambda ){Z}_{t-1}+\lambda {X}_{t},\end{eqnarray*}

where 0 < *λ* ≤ 1 is smoothing parameter and *X*_*t*_, *t* = 1, 2, 3, … isa sequence of autocorrelated observations with the starting value is the process target mean *Z*_0_ = *μ*_0_.

The respective upper and lower control limits for the EWMA chart are

(2) \begin{eqnarray*}& & UCL={\mu }_{0}+H\sigma \sqrt{ \frac{\lambda }{2-\lambda } \left[ 1-(1-\lambda )^{2i} \right] },\end{eqnarray*}

(3) \begin{eqnarray*}& & LCL={\mu }_{0}-H\sigma \sqrt{ \frac{\lambda }{2-\lambda } \left[ 1-(1-\lambda )^{2i} \right] },\end{eqnarray*}

where *H* is the width of the control limit and *σ* is the process standard deviation. The term $ \left[ 1-(1-\lambda )^{2i} \right] $ is close to 1 as *i* becomes large. Hence, the respective upper and lower control limit will approach their respective steady-state value as

(4) \begin{eqnarray*}& & UCL={\mu }_{0}+H\sigma \sqrt{ \frac{\lambda }{2-\lambda } },\end{eqnarray*}

and

(5) \begin{eqnarray*}& & LCL={\mu }_{0}-H\sigma \sqrt{ \frac{\lambda }{2-\lambda } }.\end{eqnarray*}

Later, modified EWMA control chart was introduced by Patel & Divecha (2011). They corrected the inertia problem caused by errors in the EWMA statistic by considering past changes as well as the latest change in the process. The modified EWMA chart is useful for detecting process changes in observations that are autocorrelated or independent normally distributed. Recently, Khan, Aslam & Jun (2017) proposed a new control statistic structure that developed from the modified EWMA control chart as follow:

(6) \begin{eqnarray*}& & {Z}_{t}=(1-\lambda ){Z}_{t-1}+\lambda {X}_{t}+k({X}_{t}-{X}_{t-1}),\end{eqnarray*}

where *λ* is the smoothing parameter; *X*_*t*_, *t* = 1, 2, 3, … is a sequence of autocorrelated observations; *k* is a constant; and the starting value is the process target mean *Z*_0_ = *μ*_0_. It is similar to the EWMA statistic but with the last term extended.

This chart generates a false alarm when the *Z*_*n*_ value violates the specified control limit. In general, the upper and lower control limit are respectively given by

(7) \begin{eqnarray*}& & UCL={\mu }_{0}+L\sigma \sqrt{ \frac{\lambda +2k\lambda +2{k}^{2}}{2-\lambda } },\end{eqnarray*}

and

(8) \begin{eqnarray*}& & LCL={\mu }_{0}-L\sigma \sqrt{ \frac{\lambda +2k\lambda +2{k}^{2}}{2-\lambda } },\end{eqnarray*}

where *L* and *σ* is the width of the control limit of modified EWMA procedure and process standard deviation, respectively.

### Method of evaluating ARL for moving average order *q* model

A time series is a series of data points ordered in time, and the goal of a time series analysis is usually to make a forecast of the future using time as an independent variable. A usual characteristic of a time series is autocorrelation, which is correlation between observations in the same dataset at different points in time. In other words, autocorrelated data portrays the similarity between observations as a function of the time lag between them. In a time series analysis, an MA(q) process is a common approach for modeling a univariate time series for which the error depends linearly on the current and numerous past values of the error term.

(9) \begin{eqnarray*}& & {X}_{t}=\mu +{\varepsilon }_{t}-{\theta }_{1}{\varepsilon }_{t-1}-{\theta }_{2}{\varepsilon }_{t-2}-\cdots -{\theta }_{q}{\varepsilon }_{t-q},\end{eqnarray*}

where µis the mean of the series, ε_*t*_ is a white noise process assumed to be exponentially distributed, *θ*_*i*_ is a process coefficient, and the starting value of ε_0_= *s* is given.

Let *L*(*u*) denote the ARL for an MA(q) process with exponential white noise on a modified EWMA control chart (see Appendix A1 for the proof) that there exists only one solution of the integral equation (see Appendix A2). It is obtained by deriving a Fredholm integral equation of the second kind as follows:

(10) \begin{eqnarray*}& & L(u)=1- \frac{\lambda {e}^{ \frac{(1-\lambda )u}{{\alpha }_{0} \left( \lambda +k \right) } } \left[ {e}^{- \frac{b}{{\alpha }_{0} \left( \lambda +k \right) } }-1 \right] }{\lambda {e}^{ \frac{-\mu }{{\alpha }_{0}} }\cdot {e}^{ \frac{v+ \left( \lambda {\theta }_{1}+{\theta }_{1}k \right) s+ \left( \lambda {\theta }_{2}+{\theta }_{2}k \right) {\varepsilon }_{t-2}+\cdots + \left( \lambda {\theta }_{q}+{\theta }_{q}k \right) {\varepsilon }_{t-q}}{{\alpha }_{0} \left( \lambda +k \right) } }+{e}^{- \frac{\lambda b}{{\alpha }_{0} \left( \lambda +k \right) } }-1} ,\end{eqnarray*}

with in-control process parameter *α*_0_ and out-of-control process parameter *α*_1_ > *α*_0_.

### Numerical results

The ARLs of the explicit formulas are derived using a Fredholm integral equation of the second type, those of the numerical integral equation method are approximated using the Gauss-Legendre quadrature rule with 1,000 nodes, and the control ARL is set to ARL_0_ = 500. The numerical approximation of the numerical integral equation and the exact result of the explicit formulas to measure the accuracy in the comparative study according to the relative error is defined as

(11) \begin{eqnarray*}& & \varepsilon = \frac{{|}L(u)-\hat {L}(u){|}}{L(u)} \times 100\text{%},\end{eqnarray*}

where *L*(*u*) is derived from the ARL using the explicit formulas and $\hat {L}(u)$ is an approximation of the ARL with the numerical integral equation. The numerical results are reported in Tables 1, 2 and 3.

**Table 1:** The ARL of explicit formulas and NIE method for MA(2) when *μ* = 2 and *k* = 1 on modified EWMA control chart.

*λ*	*θ*_*i*_	***b***	*δ*	**Explicit**	**NIE**	**ε**
			0.00	500.000070	500.000063	1.249 × 10^−^^6^
			0.001	344.029967	344.029963	1.098 × 10^−^^6^
			0.003	211.859210	211.859208	9.666 × 10^−^^7^
			0.005	153.059939	153.059937	9.058 × 10^−^^7^
0.05	*θ*_1_ = − 0.3	0.4528820782	0.01	90.369435	90.369435	8.348 × 10^−^^7^
	*θ*_2_ = 0.5		0.05	21.191203	21.191203	6.871 × 10^−^^7^
			0.10	10.915019	10.915019	5.891 × 10^−^^7^
			0.50	2.615077	2.615077	2.141 × 10^−^^7^
			1.00	1.693016	1.693016	8.269 × 10^−^^8^
			0.00	500.000081	500.000068	2.634 × 10^−^^6^
			0.001	334.507743	334.507737	1.996 × 10^−^^6^
			0.003	201.308260	201.308257	1.479 × 10^−^^6^
			0.005	144.002910	144.002908	1.254 × 10^−^^6^
0.1	*θ*_1_ = − 0.3	0.45905302	0.01	84.171367	84.171366	1.014 × 10^−^^6^
	*θ*_2_ = 0.5		0.05	19.612599	19.612599	6.883 × 10^−^^7^
			0.10	10.146990	10.146990	5.686 × 10^−^^7^
			0.50	2.508587	2.508587	1.953 × 10^−^^7^
			1.00	1.653220	1.653220	7.259 × 10^−^^8^
			0.00	500.000144	500.000105	7.802 × 10^−^^6^
			0.001	334.491414	334.491395	5.543 × 10^−^^6^
			0.003	201.328130	201.328122	3.721 × 10^−^^6^
			0.005	144.051692	144.051688	2.934 × 10^−^^6^
0.15	*θ*_1_ = 0.1	0.572945976	0.01	84.258340	84.258339	2.104 × 10^−^^6^
	*θ*_2_ = 0.3		0.05	19.742557	19.742557	1.116 × 10^−^^6^
			0.10	10.274933	10.274932	8.711 × 10^−^^7^
			0.50	2.592448	2.592448	2.893 × 10^−^^7^
			1.00	1.709825	1.709825	1.053 × 10^−^^7^
			0.00	500.000089	500.000024	1.309 × 10^−^^5^
			0.001	326.638522	326.638494	8.859 × 10^−^^6^
			0.003	192.985129	192.985118	5.595 × 10^−^^6^
0.2	*θ*_1_ = 0.1	0.583106542	0.005	137.017229	137.017224	4.225 × 10^−^^6^
	*θ*_2_ = 0.3		0.01	79.531204	79.531202	2.809 × 10^−^^6^
			0.05	18.554510	18.554510	1.219 × 10^−^^6^
			0.10	9.693785	9.693784	9.119 × 10^−^^7^
			0.50	2.508174	2.508174	2.751 × 10^−^^7^
			1.00	1.677336	1.677336	1.014 × 10^−^^7^

**Notes.**

Where *λ* is a smoothing parameter, *θ*_*i*_ is a process coefficient, *b* is UCL, *δ* is the shift size and ε is the relative error.

**Table 2:** The ARL of explicit formulas and NIE method for MA(3) when *μ* = 2 and *k* = 1 on modified EWMA control chart.

*λ*	*θ*_*i*_	***b***	*δ*	**Explicit**	**NIE**	**ε**
			0.00	500.000035	499.999925	2.200 × 10^−^^5^
			0.001	416.626140	416.226056	2.013 × 10^−^^5^
			0.003	312.391104	312.391049	1.777 × 10^−^^5^
	*θ*_1_ = 0.3		0.005	249.841554	249.841513	1.634 × 10^−^^5^
0.05	*θ*_2_ = 0.5	1.7145985314	0.01	166.435290	166.435266	1.437 × 10^−^^5^
	*θ*_3_ = 0.7		0.05	45.160512	45.160507	1.073 × 10^−^^5^
			0.10	23.579116	23.579114	9.196 × 10^−^^6^
			0.50	5.145761	5.145761	4.015 × 10^−^^6^
			1.00	2.933150	2.933150	1.841 × 10^−^^6^
			0.00	500.000093	499.999812	5.633 × 10^−^^5^
			0.001	412.884959	412.884760	4.831 × 10^−^^5^
			0.003	306.166728	306.166611	3.847 × 10^−^^5^
	*θ*_1_ = 0.3		0.005	243.266257	243.266177	3.265 × 10^−^^5^
0.1	*θ*_2_ = 0.5	1.790036614	0.01	160.685954	160.685914	2.495 × 10^−^^5^
	*θ*_3_ = 0.7		0.05	43.171580	43.171574	1.322 × 10^−^^5^
			0.10	22.563452	22.563449	1.031 × 10^−^^6^
			0.50	5.001808	5.001808	4.065 × 10^−^^6^
			1.00	2.881284	2.881284	1.833 × 10^−^^6^
			0.00	500.000074	500.000073	2.124 × 10^−^^6^
			0.001	270.407899	270.407899	1.293 × 10^−^^6^
			0.003	140.988209	140.988209	8.213 × 10^−^^7^
	*θ*_1_ = − 0.5		0.005	95.372295	95.372295	6.522 × 10^−^^7^
0.15	*θ*_2_ = − 0.3	0.10145919916	0.01	52.755839	52.755839	4.928 × 10^−^^7^
	*θ*_3_ = − 0.5		0.05	11.649752	11.649752	3.004 × 10^−^^7^
			0.10	6.024879	6.024879	2.324 × 10^−^^7^
			0.50	1.671266	1.671266	5.983 × 10^−^^8^
			1.00	1.247145	1.247145	0.000 × 10^−^^7^
			0.00	500.000023	500.000021	3.506 × 10^−^^6^
			0.001	260.636834	260.636833	1.964 × 10^−^^6^
			0.003	133.218384	133.218384	1.140 × 10^−^^6^
	*θ*_1_ = − 0.5		0.005	89.517777	89.517777	8.557 × 10^−^^7^
0.2	*θ*_2_ = − 0.3	0.1023254883	0.01	49.241334	49.241334	5.910 × 10^−^^7^
	*θ*_3_ = − 0.5		0.05	10.884204	10.884204	3.032 × 10^−^^7^
			0.10	5.670187	5.670187	2.293 × 10^−^^7^
			0.50	1.630976	1.630976	6.131 × 10^−^^8^
			1.00	1.234071	1.234071	0.000 × 10^−^^7^

**Notes.**

Where *λ* is a smoothing parameter, *θ*_*i*_ is a process coefficient, *b* is UCL, *δ* is the shift size and ε is the relative error.

**Table 3:** The ARL of explicit formulas and NIE method for MA(3) when *μ* = 2, *λ* = 0.1 and *k* = 5*λ*, 10*λ*, 20*λ*, 50*λ* on modified EWMA control chart.

***k***	*θ*_*i*_	***b***	*δ*	**Explicit**	**NIE**	**ε**
			0.00	500.000066	500.000043	4.561 × 10^−^^6^
			0.001	413.570468	413.570451	4.083 × 10^−^^6^
			0.003	307.119823	307.119812	3.491 × 10^−^^6^
	*θ*_1_ = − 0.3		0.005	244.081098	244.081090	3.137 × 10^−^^6^
5*λ*	*θ*_2_ = 0.7	0.3993899124	0.01	160.988028	160.988024	2.660 × 10^−^^6^
	*θ*_3_ = − 0.5		0.05	42.170082	42.170082	1.846 × 10^−^^6^
			0.10	21.358100	21.358099	1.552 × 10^−^^6^
			0.50	4.092363	4.092363	6.280 × 10^−^^7^
			1.00	2.254223	2.254223	2.573 × 10^−^^7^
			0.00	500.000057	500.000050	1.383 × 10^−^^6^
			0.001	322.652564	322.652560	1.028 × 10^−^^6^
			0.003	188.777532	188.777531	7.591 × 10^−^^7^
	*θ*_1_ = − 0.3		0.005	133.436625	133.436624	6.465 × 10^−^^7^
10*λ*	*θ*_2_ = 0.7	0.3381621032	0.01	77.030788	77.030787	5.282 × 10^−^^7^
	*θ*_3_ = − 0.5		0.05	17.693001	17.693001	3.662 × 10^−^^7^
			0.10	9.130173	9.130173	3.023 × 10^−^^7^
			0.50	2.283033	2.283033	1.007 × 10^−^^7^
			1.00	1.537883	1.537883	3.251 × 10^−^^8^
			0.00	500.000064	500.000060	7.459 × 10^−^^7^
			0.001	259.369506	259.369504	4.634 × 10^−^^7^
			0.003	132.360364	132.360364	3.136 × 10^−^^7^
	*θ*_1_ = − 0.3		0.005	88.971221	88.971221	2.615 × 10^−^^7^
20*λ*	*θ*_2_ = 0.7	0.416955807	0.01	49.057165	49.057164	2.118 × 10^−^^7^
	*θ*_3_ = − 0.5		0.05	11.092459	11.092459	1.451 × 10^−^^7^
			0.10	5.913303	5.913303	1.167 × 10^−^^7^
			0.50	1.796095	1.796095	3.341 × 10^−^^8^
			1.00	1.339172	1.339172	7.467 × 10^−^^9^
			0.00	500.000067	500.000064	5.106 × 10^−^^7^
			0.001	218.076288	218.076288	2.735 × 10^−^^7^
			0.003	102.797634	102.797634	1.760 × 10^−^^7^
	*θ*_1_ = − 0.3		0.005	67.421706	67.421706	1.455 × 10^−^^7^
50*λ*	*θ*_2_ = 0.7	0.763809721	0.01	36.459416	36.459416	1.174 × 10^−^^7^
	*θ*_1_ = − 0.5		0.05	8.314559	8.314559	7.938 × 10^−^^8^
			0.10	4.565969	4.565969	6.132 × 10^−^^8^
			0.50	1.588295	1.588295	1.889 × 10^−^^8^
			1.00	1.253079	1.253079	0.000 × 10^−^^8^

**Notes.**

Where *λ* is a smoothing parameter, *θ*_*i*_ is a process coefficient, *b* is UCL, *δ* is the shift size and ε is the relative error.

Computation of the ARL by using the explicit formulas and the numerical integral equation method on the modified EWMA control chart were carried out with a varied smoothing parameters (*λ* = 0.05, 0.10, 0.15 and 0.2); constant *k* = 1; in-control process parameter *α*_0_ = 1; out-of-control process parameter *α*_1_ = (1 + *δ*)*α*_0_, where *δ* is the shift size set as 0.001, 0.003, 0.005, 0.01, 0.05, 0.10, 0.50 or 1.00; and in-control process was ARL_0_ = 500 (Tables 1 and 2). The parameters were set as *μ* = 2; coefficients *θ*_1_ =  − 0.3, *θ*_2_ = 0.5 with *λ* = 0.05, 0.1 and *θ*_1_ = 0.1, *θ*_2_ = 0.3 with *λ* = 0.15, 0.2 for an MA(2) process; and coefficients *θ*_1_ = 0.3, *θ*_2_ = 0.5, *θ*_3_ = 0.7 with *λ* = 0.05, 0.1 and *θ*_1_ =  − 0.5, *θ*_2_ =  − 0.3, *θ*_3_ =  − 0.5 with *λ* = 0.15, 0.2 for an MA(3) process. The results indicate that when the smoothing parameter was increased, the value of ARL_1_ was reduced.

The results in Tables 1 and 2 show that the analytically explicit expression of the ARL was in excellent agreement with the approximated ARL obtained from the numerical integral equation (NIE) method. The computational time for the numerical integral equation method was around 21 and 23 s for the MA(2) and MA(3) processes, respectively, while the explicit formulas required a computational time of less than one second for both.

Table 3 reports the ARL values obtained by using the explicit formulas and numerical integral equation method. The parameters were set as *μ* = 2; coefficient parameters *θ*_1_ =  − 0.3, *θ*_2_ = 0.7, *θ*_3_ =  − 0.5 for MA(3) process; *λ* = 0.1; and *k* was varies as 5*λ*, 10*λ*, 20*λ*, or 50*λ*. The results revealed that when the constant *k* was large, ARL_1_ was reduced.

The performances of the standard and modified EWMA control charts were also compared. These were obtained by the explicit expression when varying *λ* (0.05 and 0.10) for both control charts, as reported in Tables 4 and 5. The observations were from the MA(2) and MA(3) processes with *θ*_1_ =  − 0.1, *θ*_2_ =  − 0.3, and *θ*_1_ = 0.7, *θ*_2_ = 0.7, *θ*_3_ =  − 0.1, respectively, for *μ* = 2, *k* = 1, and ARL_0_ = 500. The last row is the relative mean index (*RMI*) defined as

(12) \begin{eqnarray*}& & RMI= \frac{1}{n} \sum _{i=1}^{n} \left[ \frac{AR{L}_{{\delta }_{i}}-AR{L}_{{\delta }_{i}}^{\text{smallest}}}{AR{L}_{{\delta }_{i}}^{\text{smallest}}} \right] ,\end{eqnarray*}

where *ARL*_*δ*_*i*__denotes the ARLs of the EWMA and modified EWMA control charts obtained via the explicit formulas for each shift size and $AR{L}_{{\delta }_{i}}^{\text{smallest}}$ denotes the smallest of the ARLs for each shift size.

**Table 4:** Comparison ARL for MA(2) and MA(3) when *μ* = 2, (*θ*_1_, *θ*_2_) = (−0.1,−0.3), (*θ*_1_, *θ*_2_, *θ*_3_) = (0.7, 0.7, −0.1) and *k*= 1 on EWMA and modified EWMA control charts using by explicit formulas.

**Shift size**	MA(2)			
	***λ* = 0.05**		***λ* = 0.1**	
**(*δ*)**	**EWMA** (*h*= 1.540947 × 10^−^^6^)	**Modified** (*b*= 0.247244692)	**EWMA** (*h*= 9.722515 × 10^−^^2^)	**Modified** (*b*= 0.2494786708)
0.00	500.000084	500.000051	500.000053	500.000045
0.001	491.302902	322.103642	496.493518	311.471395
0.003	474.409028	188.165212	489.573629	177.579957
0.005	458.159230	132.886351	482.775802	124.203548
0.01	420.179693	76.597597	466.298614	70.932841
0.05	216.581889	17.452224	357.212201	16.095125
0.10	101.304137	8.935028	262.720549	8.288535
0.30	9.326784	3.244020	96.241953	3.076188
0.50	2.314971	2.182431	45.489101	2.100245
1.00	1.062006	1.475103	13.178794	1.446133
2.00	1.002599	1.189396	3.916046	1.179423
***RMI***	3.266348	0.057529	12.408089	0.000000
**Shift size**	MA(3)			
	***λ* = 0.15**		***λ* = 0.2**	
**(*δ*)**	**EWMA** (*h*= 0.26180448)	**Modified** (*b*= 1.495885499)	**EWMA** (*h*= 0.48260591)	**Modified** (*b*= 1.552310613)
0.00	500.000023	500.000016	500.000059	500.000012
0.001	497.068024	389.027511	497.491338	384.835986
0.003	491.267689	269.482277	492.501520	263.538348
0.005	485.551042	206.178820	487.548819	200.434640
0.01	471.616385	129.968083	475.331533	125.474506
0.05	376.298194	33.106525	386.389784	31.756669
0.10	288.608113	17.342524	297.158447	16.662591
0.30	117.137775	6.311771	113.435186	6.117180
0.50	58.046820	4.096353	53.536263	3.995715
1.00	17.466862	2.475941	15.918420	2.438029
2.00	5.133009	1.705112	5.033017	1.691792
***RMI***	6.988635	0.000000	7.082964	0.000000

**Notes.**

Where *λ* is a smoothing parameter, *b* is UCL of the modified chart, and *h* is UCL of the EWMA chart.

**Table 5:** Comparison ARL for MA(2) observations for PM2.5 in Thailand when *μ* = 51.163, (*θ*_1_, *θ*_2_) = (−0.723, −0.380) and *α*_0_ = 8.90 on EWMA and modified EWMA control charts using by explicit formulas.

**Shift size**	***λ* = 0.05**		***λ* = 0.1**	
**(*δ*)**	**EWMA** (*h*= 4.2219 × 10^−^^5^)	**Modified** (*b*= 0.02922099)	**EWMA** (*h*= 0.041728)	**Modified** (*b*= 0.03046693)
0.00	500.058819	500.062160	500.040155	500.049594
0.001	499.135917	372.303952	499.464575	371.313138
0.003	497.295828	246.500852	498.315774	245.210289
0.005	495.463334	184.318545	497.170110	183.121388
0.01	490.915098	113.156303	494.319608	112.261092
0.05	456.167456	28.053250	472.201199	27.791251
0.10	416.548214	14.711725	446.185321	14.575755
0.30	292.467926	5.402048	357.803981	5.358236
0.50	208.450137	3.507836	289.541426	3.483141
1.00	94.976087	2.098796	176.853408	2.088303
2.00	24.817914	1.428039	75.347531	1.424175
***RMI***	22.115534	0.000000	33.559477	0.000000
**Shift size**	***λ* = 0.15**		***λ* = 0.2**	
**(*δ*)**	**EWMA** (*h*= 0.211034)	**Modified** (*b*= 0.03171369)	**EWMA** (*h*= 0.305241)	**Modified** (*b*= 0.03296119)
0.00	500.037390	500.045701	500.042301	500.068733
0.001	499.529929	370.409082	499.539394	369.591967
0.003	498.516876	244.034940	498.535421	242.964923
0.005	497.506311	182.033100	497.533895	181.042240
0.01	494.990736	111.449184	495.040738	110.710551
0.05	475.411216	27.554328	475.631720	27.339135
0.10	452.240112	14.452853	452.652967	14.341252
0.30	372.239132	5.318641	373.229915	5.282691
0.50	308.827934	3.460822	310.167476	3.440555
1.00	199.849984	2.078817	210.495325	2.070201
2.00	93.953526	1.420680	95.355765	1.417504
***RMI***	37.060241	0.000000	37.933433	0.000000

**Notes.**

Where *λ* is a smoothing parameter, *b* is UCL of the modified chart, and *h* is UCL of the EWMA chart.

The results in Table 4 show that when *λ* = 0.05, the performance of the modified EWMA control chart was better than the standard one for shift sizes of 0.001, 0.003, 0.005, 0.01, 0.05, 0.10 and 0.30, whereas for shift size of 0.50, 1.00, and 2.00, the small *RMI* of the modified EWMA chart was 0.057529 while that the *RMI* of the EWMA control chart was 3.266348. When *λ* = 0.1,  0.15 and 0.2, the modified EWMA control chart was more powerful than the standard one for all cases of shift size with the zero *RMI*. The results indicate that overall, the modified EWMA control chart was better than the standard one at detecting process changes.

### Application of the modified EWMA chart

PM2.5 and CO gas air pollutants are being constantly emitted, which is likely to increase over time in the winter and summer seasons. When the levels of these air pollutants are high (>50 µg/m^3^ for PM2.5 (https://en.wikipedia.org/wiki/Air_quality_guideline) and >10,000 ppm for CO (https://www.airqualitynow.eu/download/CITEAIR-Comparing_Urban_Air_Quality_across_Borders.pdf)), the quality of the ambient air is unhealthy to humans. Increasing PM2.5 concentration can lead to coughing, breathing difficulties, and eye irritation and can be deadly to humans.

Table 5 contains a comparison of the ARLs of the modified and standard EWMA control charts obtained via the explicit formulas. PM2.5 and CO measurements were taken every day in January and May, respectively, 2020 by the Pollution Control Department, Thailand. There were small and abrupt changes in the PM2.5 and CO level data in the Din Daeng district of Bangkok (where there is a high volume of traffic) from measurements near a busy road (Chuersuwan et al., 2008). The PM2.5 and CO air pollution level data were tested for autocorrelation in the observations. The Box-Jenkins technique was applied to the two air pollution datasets to determine whether they fit forecast time series data models. The models with the lowest Akaike Information Criterion (AIC) and Bayesian Information Criterion (BIC) values were considered as optimal. Moreover, *t*-test statistics proved that the two datasets were autocorrelated. The parameter values for the MA(1) and MA(2) processes were fitted and provided 51.163 for the mean and -0.723 and -0.380 for the coefficients, respectively. The PM2.5 level was found to be significant for the MA(2) process.

The efficiency of the modified EWMA procedure was also emphasized by its performance with the CO level data for the Din Daeng district, Bangkok, Thailand. Table 6 displays the ARLs of the modified and traditional EWMA control charts. The explicit formulas were used for measuring the ARLs of the CO gas level. The data were collected every day in May 2020. The analysis for an MA autocorrelated process resulted in a mean of 1.198 and −0.662, −0.479, and −0.495 for the coefficients of the MA(1), MA(2), and MA(3) processes. The results of the PM2.5 and CO air pollutant data indicate that the modified EWMA control chart was more effective than the standard one for detecting small shifts, and so confirms that it is excellent for monitoring unusual observations with undesirable values in a timely manner for all cases of exponential smoothing parameter.

**Table 6:** Comparison ARL for MA(3) observations for CO gas in Thailand when *μ* = 1.198, (*θ*_1_, *θ*_2_, *θ*_3_) = (−0.662, −0.479, −0.495) and *α*_0_ = 0.1226 on EWMA and modified EWMA control charts using by explicit formulas.

**Shift size**	***λ* = 0.05**		***λ* = 0.1**	
**(*δ*)**	**EWMA** (*h*= 2.15351 × 10^−^^10^)	**Modified** (*b*= 5.6968 × 10^−^^10^)	**EWMA** (*h*= 4.97638 × 10^−^^9^)	**Modified** (*b*= 5.00361 × 10^−^^10^)
0.00	500.018859	500.018440	500.048462	500.049041
0.001	410.684518	12.373073	418.900893	11.525787
0.003	279.691311	4.269468	296.482942	4.020852
0.005	192.837824	2.664904	212.155442	2.537072
0.01	80.144470	1.544761	96.189807	1.501932
0.05	1.406011	1.004560	1.824931	1.004121
0.10	1.007555	1.000146	1.022682	1.000130
0.30	1.000009	1.000000	1.000051	1.000000
0.50	1.000001	1.000000	1.000005	1.000000
1.00	1.000000	1.000000	1.000000	1.000000
2.00	1.000000	1.000000	1.000000	1.000000
***RMI***	21.935199	0.000000	25.458726	0.000000
**Shift size**	***λ* = 0.15**		***λ* = 0.2**	
**(*δ*)**	**EWMA** (*h*= 8.7735 × 10^−^^6^)	**Modified** (*b*= 4.45335 × 10^−^^10^)	**EWMA** (*h*= 9.2341 × 10^−^^4^)	**Modified** (*b*= 4.009478 × 10^−^^10^)
0.00	500.061271	500.067195	500.027847	500.029982
0.001	443.510830	10.806701	459.400446	10.190344
0.003	350.844729	3.810433	389.296622	3.630480
0.005	279.559120	2.428984	331.547146	2.336624
0.01	163.232107	1.465789	226.523241	1.434960
0.05	7.394604	1.003757	23.667633	1.003452
0.10	1.543142	1.000116	4.865620	1.000105
0.30	1.007718	1.000000	1.171529	1.000000
0.50	1.001440	1.000000	1.048075	1.000000
1.00	1.000237	1.000000	1.011600	1.000000
2.00	1.000061	1.000000	1.003766	1.000000
***RMI***	36.248876	0.000000	47.475014	0.000000

**Notes.**

Where *λ* is a smoothing parameter, *b* is UCL of the modified chart, and *h* is UCL of the EWMA chart.

**Figure 1:**
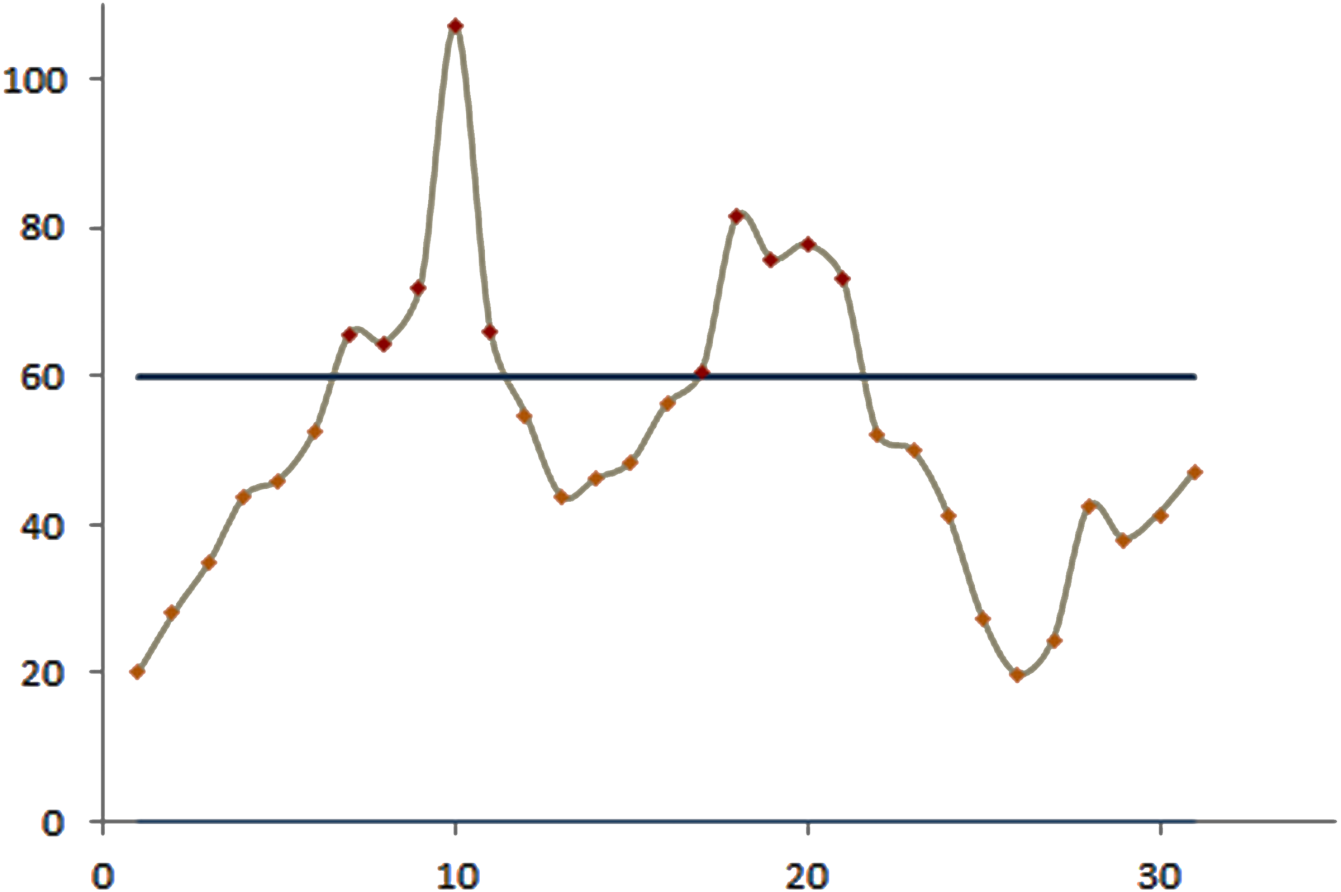
The process detecting of PM2.5 level observations of modified EWMA control chart.

**Figure 2:**
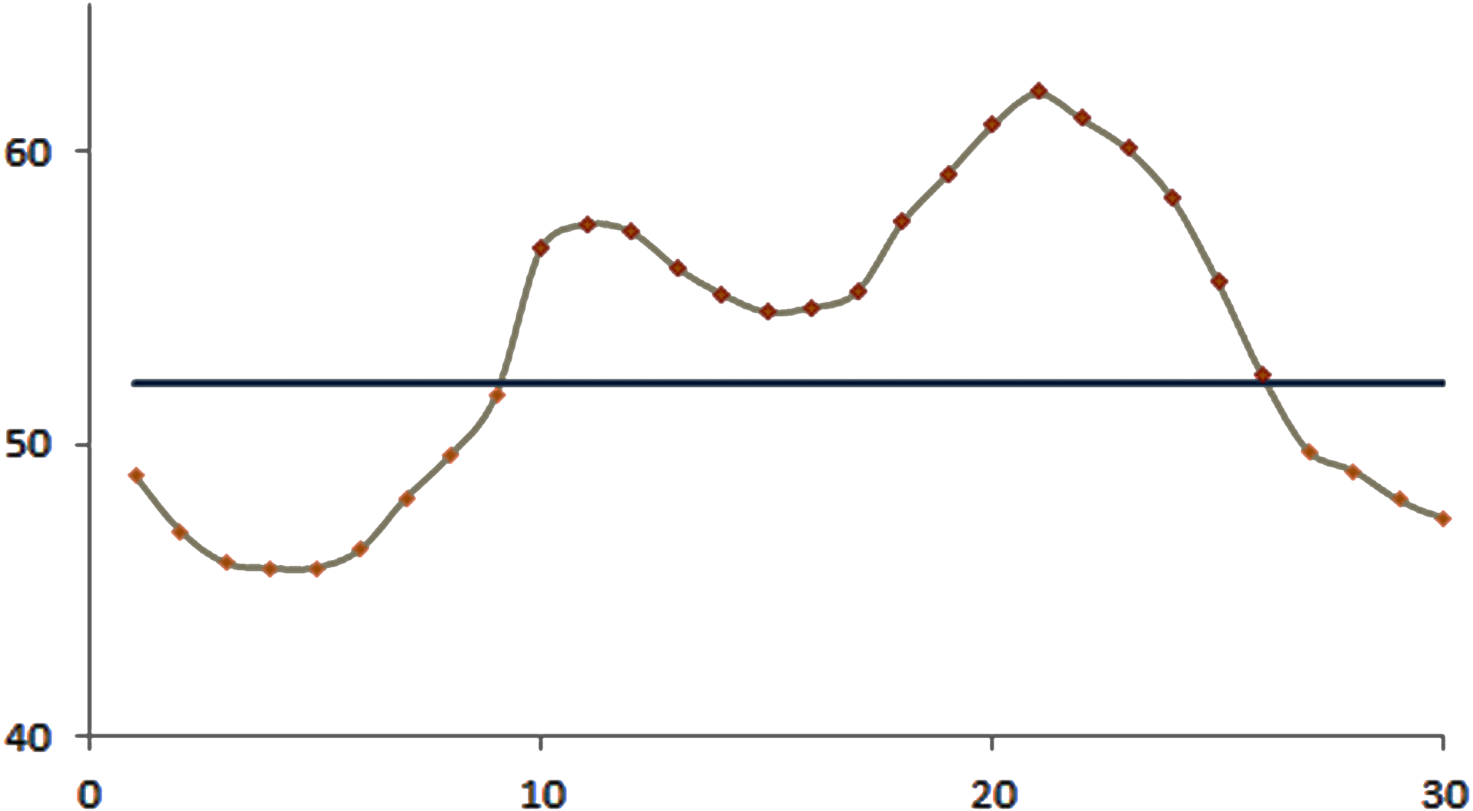
The process detecting of PM2.5 level observations of the EWMA control chart.

**Figure 3:**
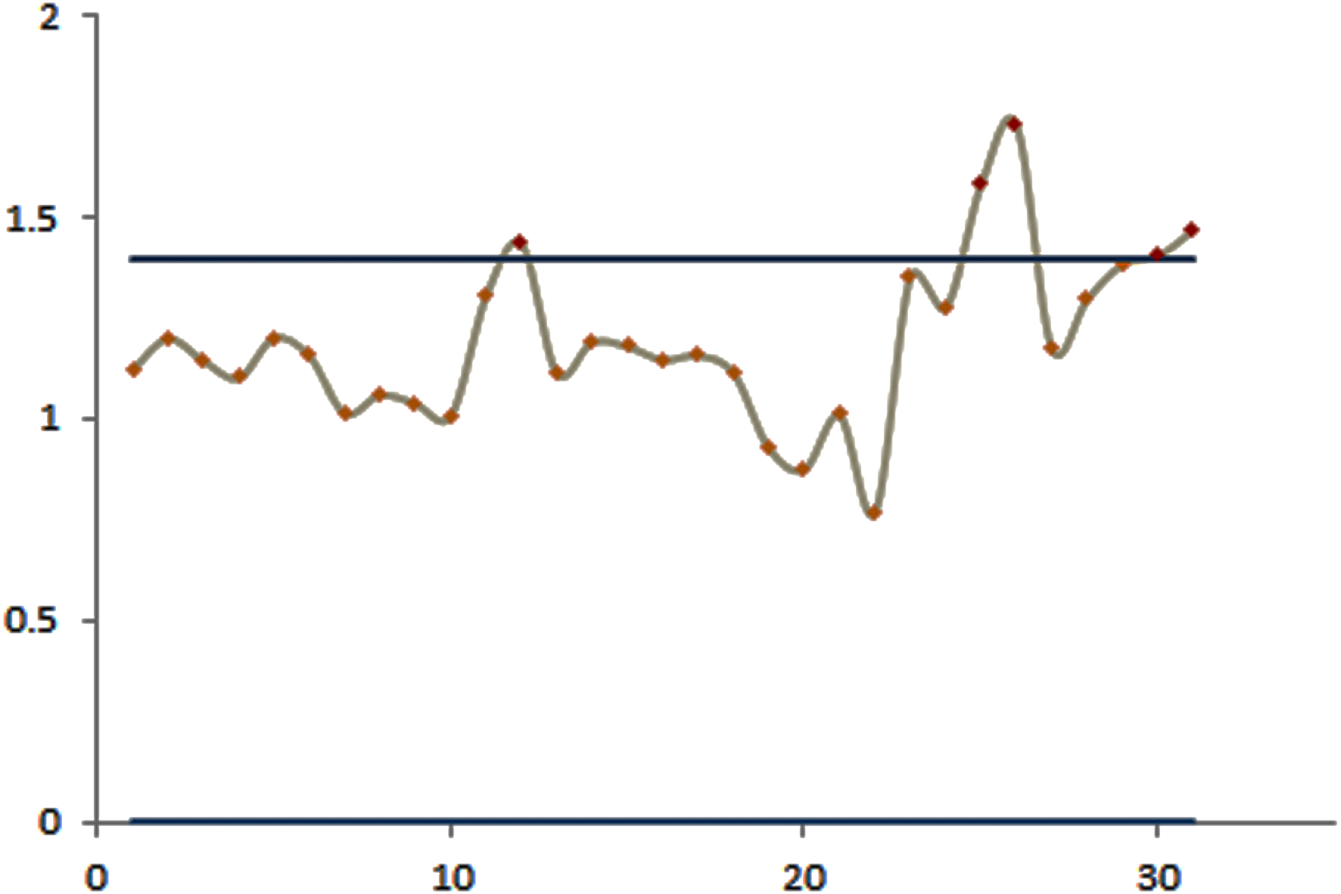
The process detecting of CO gas level observations of the modified EWMA control chart.

**Figure 4:**
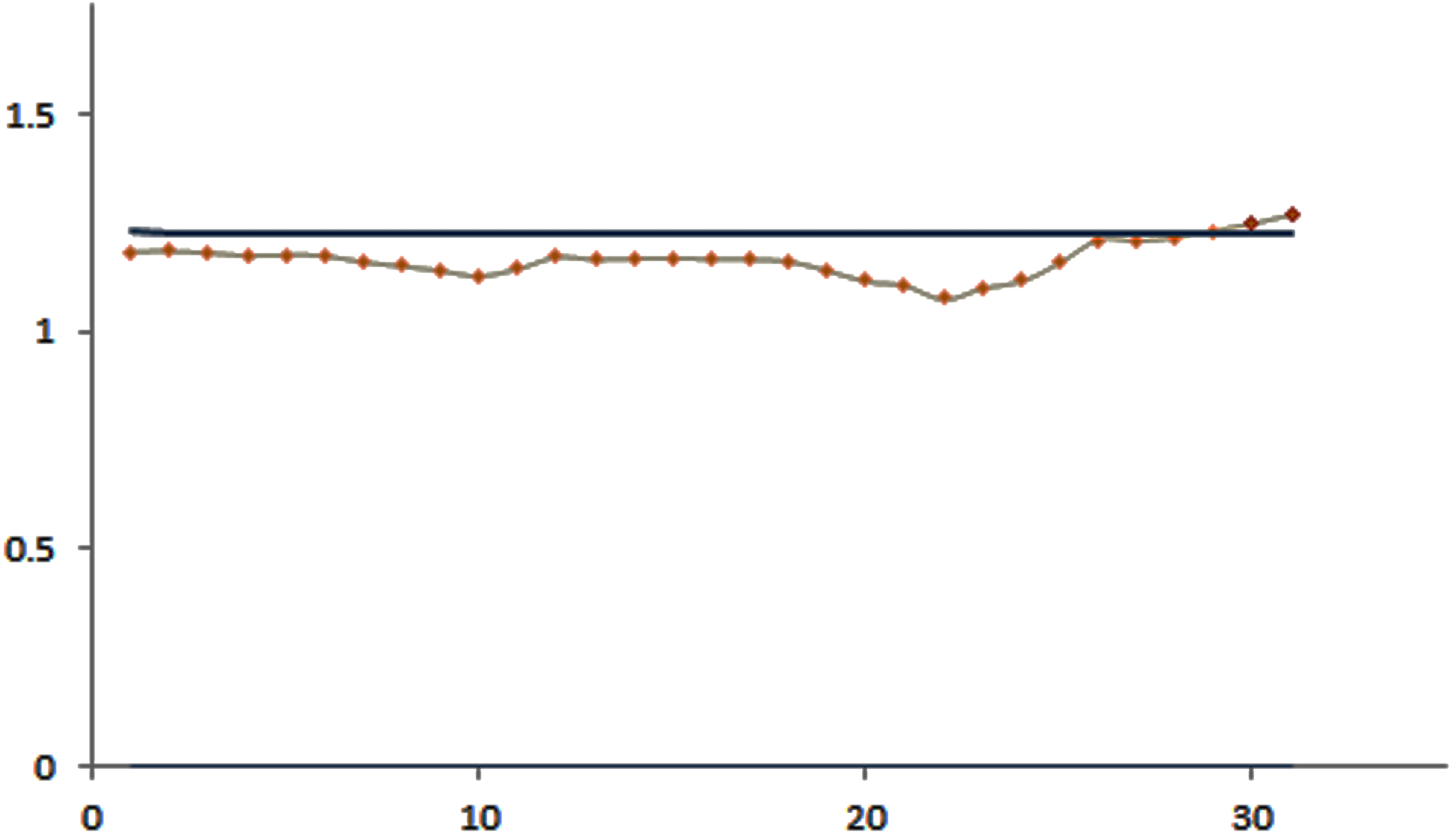
The process detecting of CO gas level observations of the EWMA control chart.

The efficacy of the control charts was visualized by plotting graphs to showcase the effective results obtained from the proposed procedure in a comparative study. Figure 1 shows that the modified EWMA control chart detected upper PM2.5 shifts at the 7th to 11th and 17th to 21st observations. On the other hand, the standard EWMA control chart only detected shifts at the 10th to 26th observations, as illustrated in Fig. 2. Figure 3 exhibits that the modified EWMA chart detected upper CO level shifts at the 12th, 25th to 26th, and 30th to 31st observations. On the contrary, the original EWMA chart only detected upper CO level shifts at the 30th to 31st observations, as shown in Fig. 4.

The modified EWMA control chart detected the upper change in PM2.5 level at the 7th observation (i.e., the 7th January), which marked the beginning of extreme changes in PM2.5 emissions at the upper level. Meanwhile, the standard EWMA control chart detected the change at the 10th observation (i.e., the 10th January). Although the CO gas level emissions were low and harmless to the human body, the performance of the modified EWMA control chart for detecting the change in CO gas emissions was exemplary.

## Discussion

The findings reveal that the modified EWMA control chart performed well for the case of smoothing parameter is greater than or equal to 0.1 due to the *RMI* of the modified EWMA chart being less than the *RMI* of the EWMA chart.

When applied to real data, the modified EWMA control chart performed excellently for detecting shifts in the PM2.5 and CO pollution levels in all cases of smoothing parameter value. The smoothing parameter value of 0.1 is recommended in applications using the modified EWMA control chart. It is a good choice as it is easier to employ and performed better than the original EWMA control chart in all situations tested.

## Conclusions

The exact ARL was provided by deriving explicit formulas that saved significantly on computational time. Therefore, it is an excellent alternative for evaluating the ARL as a measure of the effectiveness of the modified EWMA control chart. The technique showed good aptitude in monitoring and detecting small process shifts, as illustrated by changes in PM2.5 and CO gas levels examples comprising autocorrelated observations fitted to MA(2) and MA(3) models with exponential white noise. The empirical ARL shows that a smoothing parameter value of 0.1 to 0.2 supported the modified EWMA control chart far better than the EWMA control chart for all cases. Therefore, determination of the correct smoothing parameter of the chart should not be disregarded.

##  Supplemental Information

10.7717/peerj.10467/supp-1 Supplemental Information 1 Code

10.7717/peerj.10467/supp-2 Supplemental Information 2 Raw data for application of modified EWMA and EWMA control chart performances

## Appendix A1: Derivation ARL for MA(*q*) process

According to an MA(*q*) process in the Eq. (9) as follows:

\begin{eqnarray*}& & {X}_{t}=\mu +{\varepsilon }_{t}-{\theta }_{1}{\varepsilon }_{t-1}-{\theta }_{2}{\varepsilon }_{t-2}-\cdots -{\theta }_{q}{\varepsilon }_{t-q}. (9) \end{eqnarray*}

Therefore, the modified EWMA statistic for an MA(q) model can be written as

(13) \begin{eqnarray*}{Z}_{t}& =& \left( 1-\lambda \right) {Z}_{t-1}-{X}_{t-1}+ \left( \lambda +k \right) {\varepsilon }_{t}+ \left( \lambda +k \right) \mu - \left( \lambda {\theta }_{1}+{\theta }_{1}k \right) {\varepsilon }_{t-1}\nonumber\\\displaystyle & & - \left( \lambda {\theta }_{2}+{\theta }_{2}k \right) {\varepsilon }_{t-2}-\cdots - \left( \lambda {\theta }_{q}+{\theta }_{q}k \right) {\varepsilon }_{t-q},\end{eqnarray*}

where *t* = 1,2,3,…, the initial value in the process mean *Z*_0_ = *u*, *X*_0_ = *v*, ε_0_ = *s*, and we use one side of the control limit (i.e., *LCL* = 0 and *UCL* = b). Thus, we can obtain

(14) \begin{eqnarray*}& & {Z}_{1}=(1-\lambda )u-v+ \left( \lambda +k \right) {\varepsilon }_{1}+ \left( \lambda +k \right) \mu - \left( \lambda {\theta }_{1}+{\theta }_{1}k \right) s-\cdots - \left( \lambda {\theta }_{q}+{\theta }_{q}k \right) {\varepsilon }_{1-q}.\end{eqnarray*}

If *X*_1_ causes the out-of-control state for *Z*_1_, then $(1-\lambda )u-v+ \left( \lambda +k \right) {\varepsilon }_{1}+ \left( \lambda +k \right) \mu - \left( \lambda {\theta }_{1}+{\theta }_{1}k \right) s-\cdots - \left( \lambda {\theta }_{q}+{\theta }_{q}k \right) {\varepsilon }_{1-q}\gt b$ or $(1-\lambda )u-v+ \left( \lambda +k \right) {\varepsilon }_{1}+ \left( \lambda +k \right) \mu - \left( \lambda {\theta }_{1}+{\theta }_{1}k \right) s-\cdots - \left( \lambda {\theta }_{q}+{\theta }_{q}k \right) {\varepsilon }_{1-q}\lt 0$.

If *X*_1_ causes the in-control state for *Z*_1_, then $0\lt (1-\lambda )u-v+ \left( \lambda +k \right) {\varepsilon }_{1}+ \left( \lambda +k \right) \mu - \left( \lambda {\theta }_{1}+{\theta }_{1}k \right) s-\cdots - \left( \lambda {\theta }_{q}+{\theta }_{q}k \right) {\varepsilon }_{t-q}\lt b$.

This can be written in the form

$ \frac{-(1-\lambda )u+v- \left( \lambda +k \right) \mu + \left( \lambda {\theta }_{1}+{\theta }_{1}k \right) s+\cdots + \left( \lambda {\theta }_{q}+{\theta }_{q}k \right) {\varepsilon }_{t-q}}{\lambda +k} \lt {\varepsilon }_{1}\lt \frac{b-(1-\lambda )u+v- \left( \lambda +k \right) \mu + \left( \lambda {\theta }_{1}+{\theta }_{1}k \right) s+\cdots + \left( \lambda {\theta }_{q}+{\theta }_{q}k \right) {\varepsilon }_{t-q}}{\lambda +k} .$

The probability that ε_1_ satisfies the bounds mentioned above for probability distribution function ε_*t*_ is given as follows:

$P \left( \frac{-(1-\lambda )u+v- \left( \lambda +k \right) \mu + \left( \lambda {\theta }_{1}+{\theta }_{1}k \right) s+\cdots + \left( \lambda {\theta }_{q}+{\theta }_{q}k \right) {\varepsilon }_{t-q}}{\lambda +k} \lt {\varepsilon }_{1} \right. $ $ \left. \lt \frac{b-(1-\lambda )u+v- \left( \lambda +k \right) \mu + \left( \lambda {\theta }_{1}+{\theta }_{1}k \right) s+\cdots + \left( \lambda {\theta }_{q}+{\theta }_{q}k \right) {\varepsilon }_{t-q}}{\lambda +k} \right) $ $=\int \nolimits _{ \frac{-(1-\lambda )u+v- \left( \lambda +k \right) \mu + \left( \lambda {\theta }_{1}+{\theta }_{1}k \right) s+\cdots + \left( \lambda {\theta }_{q}+{\theta }_{q}k \right) {\varepsilon }_{t-q}}{\lambda +k} }^{ \frac{b-(1-\lambda )u+v- \left( \lambda +k \right) \mu + \left( \lambda {\theta }_{1}+{\theta }_{1}k \right) s+\cdots + \left( \lambda {\theta }_{q}+{\theta }_{q}k \right) {\varepsilon }_{t-q}}{\lambda +k} }f(y)dy$.

According to the method of Champ & Rigdon (1991), let *L*(*u*) denote the ARL on a modified EWMA chart for an MA(q) process. We can write the integral equation in the form

(15) \begin{eqnarray*}L(u)=1+\int \nolimits \nolimits _{ \frac{-(1-\lambda )u+v- \left( \lambda +k \right) \mu + \left( \lambda {\theta }_{1}+{\theta }_{1}k \right) s+\cdots + \left( \lambda {\theta }_{q}+{\theta }_{q}k \right) {\varepsilon }_{1-q}}{\lambda +k} }^{ \frac{b-(1-\lambda )u+v- \left( \lambda +k \right) \mu + \left( \lambda {\theta }_{1}+{\theta }_{1}k \right) s+\cdots + \left( \lambda {\theta }_{q}+{\theta }_{q}k \right) {\varepsilon }_{1-q}}{\lambda +k} }L \left[ \begin{array}{@{}l@{}} \displaystyle (1-\lambda )u-v+ \left( \lambda +k \right) y+ \left( \lambda +k \right) \mu -\\ \displaystyle \left( \lambda {\theta }_{1}+{\theta }_{1}k \right) s-\cdots - \left( \lambda {\theta }_{q}+{\theta }_{q}k \right) {\varepsilon }_{t-q} \end{array} \right] \nonumber\\\displaystyle \chskip[4pc]f(y)dy.\end{eqnarray*}

By changing the integral variable:

$g=(1-\lambda )u-v+ \left( \lambda +k \right) y+ \left( \lambda +k \right) \mu - \left( \lambda {\theta }_{1}+{\theta }_{1}k \right) s- \left( \lambda {\theta }_{2}+{\theta }_{2}k \right) {\varepsilon }_{t-2}-\cdots - \left( \lambda {\theta }_{q}+{\theta }_{q}k \right) {\varepsilon }_{t-q}$,

we obtain

(16) \begin{eqnarray*}L(u)=1+ \frac{1}{\lambda +k} \int \nolimits \nolimits _{0}^{b}L(g)f\nonumber\\\displaystyle \quad \left( \frac{g-(1-\lambda )u+v+ \left( \lambda {\theta }_{1}+{\theta }_{1}k \right) s+ \left( \lambda {\theta }_{2}+{\theta }_{2}k \right) {\varepsilon }_{t-2}+\cdots + \left( \lambda {\theta }_{q}+{\theta }_{q}k \right) {\varepsilon }_{t-q}}{\lambda +k} -\mu \right) dg.\nonumber\\\displaystyle \end{eqnarray*}

In this study, we define ε_*t*_ is a white noise process and assumed that it is exponentially distributed with parameter *α*. Therefore, the *L*(*u*) is a Fredholm integral equation of the second kind as follow:

(17) \begin{eqnarray*}& & L(u)=1+ \frac{1}{\lambda +k} \int \nolimits \nolimits _{0}^{b}L(g) \frac{1}{\alpha } {e}^{- \frac{g-(1-\lambda )u+v+ \left( \lambda {\theta }_{1}+{\theta }_{1}k \right) s+ \left( \lambda {\theta }_{2}+{\theta }_{2}k \right) {\varepsilon }_{t-2}+\cdots + \left( \lambda {\theta }_{q}+{\theta }_{q}k \right) {\varepsilon }_{t-q}}{\alpha (\lambda +k)} + \frac{\mu }{\alpha } }dg\end{eqnarray*}

which becomes

(18) \begin{eqnarray*}& & L(u)=1+ \frac{{e}^{ \frac{(1-\lambda )u-v- \left( \lambda {\theta }_{1}+{\theta }_{1}k \right) s- \left( \lambda {\theta }_{2}+{\theta }_{2}k \right) {\varepsilon }_{t-2}+\cdots + \left( \lambda {\theta }_{q}+{\theta }_{q}k \right) {\varepsilon }_{t-q}}{\alpha \left( \lambda +k \right) } + \frac{\mu }{\alpha } }}{\alpha \left( \lambda +k \right) } \int \nolimits \nolimits _{0}^{b}L(g)\cdot {e}^{- \frac{g}{\alpha \left( \lambda +k \right) } }dg.\end{eqnarray*}

Suppose that

\begin{eqnarray*}& & C(u)={e}^{ \frac{(1-\lambda )u-v- \left( \lambda {\theta }_{1}+{\theta }_{1}k \right) s- \left( \lambda {\theta }_{2}+{\theta }_{2}k \right) {\varepsilon }_{t-2}+\cdots + \left( \lambda {\theta }_{q}+{\theta }_{q}k \right) {\varepsilon }_{t-q}}{\alpha \left( \lambda +k \right) } + \frac{\mu }{\alpha } }, 0\leq u\leq b \end{eqnarray*}

and

\begin{eqnarray*}& & D=\int \nolimits \nolimits _{0}^{b}L(g)\cdot {e}^{- \frac{g}{\alpha \left( \lambda +k \right) } }dg, \text{where}Disaconstant. \end{eqnarray*}

Thus, we can obtain

(19) \begin{eqnarray*}& & L(u)=1+ \frac{C(u)}{\alpha \left( \lambda +k \right) } D.\end{eqnarray*}

The ARL for an MA(q) process on a modified EWMA control chart is obtained by deriving a Fredholm integral equation of the second kind as follows:

\begin{eqnarray*}& & L(u)=1- \frac{\lambda {e}^{ \frac{(1-\lambda )u}{{\alpha }_{0} \left( \lambda +k \right) } } \left[ {e}^{- \frac{b}{{\alpha }_{0} \left( \lambda +k \right) } }-1 \right] }{\lambda {e}^{ \frac{-\mu }{{\alpha }_{0}} }\cdot {e}^{ \frac{v+ \left( \lambda {\theta }_{1}+{\theta }_{1}k \right) s+ \left( \lambda {\theta }_{2}+{\theta }_{2}k \right) {\varepsilon }_{t-2}+\cdots + \left( \lambda {\theta }_{q}+{\theta }_{q}k \right) {\varepsilon }_{t-q}}{{\alpha }_{0} \left( \lambda +k \right) } }+{e}^{- \frac{\lambda b}{{\alpha }_{0} \left( \lambda +k \right) } }-1} , (10) \end{eqnarray*}

with in-control process parameter *α*_0_ and out-of-control process parameter *α*_1_ > *α*_0_.

## Appendix A2: Existence and Uniqueness of ARLs

The Banach’s Fixed-point Theorem is used to show the exists and a uniqueness of the solution for ARL using the integral equation for the explicit formulas.

Let *M* ≠ ∅ be a complete metric space and *d*:*M* × *M* → *R*.*d* is a distance function on *M* such that the following axioms hold.

1. *d*(*x*, *y*) ≥ 0 for all *x*, *y* ∈ *M*

2. *d*(*x*, *y*) = 0 if and only if *x* = *y*

3. *d*(*x*, *y*) = *d*(*y*, *x*) ↔*x* = *y* for all *x*, *y* ∈ *M*

4. *d*(*x*, *y*) ≤ *d*(*x*, *z*) + *d*(*z*, *y*) for all *x*, *y*, *z* ∈ *M*.

Since $ \left( M,\mathrm{d} \right) $ is a complete metric space, it denoted the space of all continuous function on [0, *b*] with the norm ${ \left\| \cdot \right\| }_{\infty }=\sup _{u\in I} \left\vert L(u) \right\vert $ and every Cauchy’s sequence *L*_*n*__*n*≥0_ for a point of *M*converges to a point *L*_0_ ∈ [0, *b*]. In this case, let *T* be an operation in the class of all continuous functions defined by,

(20) \begin{eqnarray*}& & T \left( L(u) \right) =1+ \frac{1}{\lambda +k} \int \nolimits \nolimits _{0}^{b}L(g) \frac{1}{\alpha } {e}^{- \frac{g-(1-\lambda )u+v+ \left( \lambda {\theta }_{1}+{\theta }_{1}k \right) s+ \left( \lambda {\theta }_{2}+{\theta }_{2}k \right) {\varepsilon }_{t-2}+\cdots + \left( \lambda {\theta }_{q}+{\theta }_{q}k \right) {\varepsilon }_{t-q}}{\alpha (\lambda +k)} + \frac{\mu }{\alpha } }dg.\end{eqnarray*}

By Banach’s Fixed-point Theorem, if an operator *T* is a contraction, then the fixed-point equation *T*(*L*(*u*)) = *L*(*u*) has a unique solution. The Eq. (20) exists and has a unique solution according to the following the theorem.

**Theorem 1.** (Banach’s Fixed-point Theorem)

Let $ \left( M,\mathrm{d} \right) $ be a complete metric space and *T*:*M* → *M* be a contraction mapping with contraction constant *c* ∈ [0, 1) such that $ \left\| T({L}_{1})-T({L}_{2}) \right\| \leq c \left\| {L}_{1}-{L}_{2} \right\| $ for all *L*_1_, *L*_2_ ∈ *M*. Subsequently, there exists a unique *L*(.) ∈ *X* such that $T \left( L(u) \right) =L(u)$ (Sofonea, Han & Shillor, 2006).

***Proof***. The inequality

$ \left\| T({L}_{1})-T({L}_{2}) \right\| \leq c \left\| {L}_{1}-{L}_{2} \right\| $for all *L*_1_, *L*_2_ ∈ [0, *b*] with 0 ≤ *c* < 1.

Consider Eq. (14),

$ \left\| T({L}_{1})-T({L}_{2}) \right\| =\sup _{u\in [0,b]} \left\vert L(u) \right\vert $ $=\sup _{u\in [0,b]} \left\vert \left( {L}_{1}(g)-{L}_{2}(g) \right) \frac{1}{\lambda +k} \int \nolimits _{0}^{b}L(g) \frac{1}{\alpha } \right. $ $ \left. {e}^{- \frac{g-(1-\lambda )u+v+ \left( \lambda {\theta }_{1}+{\theta }_{1}k \right) s+ \left( \lambda {\theta }_{2}+{\theta }_{2}k \right) {\varepsilon }_{t-2}+\cdots + \left( \lambda {\theta }_{q}+{\theta }_{q}k \right) {\varepsilon }_{t-q}}{\alpha (\lambda +k)} + \frac{\mu }{\alpha } }dg \right\vert $ $\leq \sup _{u\in [0,b]} \left\vert { \left\| {L}_{1}-{L}_{2} \right\| }_{\infty } \frac{-\alpha \left( \lambda +k \right) }{\alpha \left( \lambda +k \right) } {e}^{ \frac{(1-\lambda )u-v- \left( \lambda {\theta }_{1}+{\theta }_{1}k \right) s- \left( \lambda {\theta }_{2}+{\theta }_{2}k \right) {\varepsilon }_{t-2}-\cdots - \left( \lambda {\theta }_{q}+{\theta }_{q}k \right) {\varepsilon }_{t-q}}{\alpha (\lambda +k)} + \frac{\mu }{\alpha } } \left[ {e}^{ \frac{-b}{\alpha \left( \lambda +k \right) } }-1 \right] \right\vert $

$={ \left\| {L}_{1}-{L}_{2} \right\| }_{\infty } \left\vert 1-{e}^{ \frac{-b}{\alpha \left( \lambda +k \right) } } \right\vert \sup _{u\in [0,b]} \left\vert {e}^{ \frac{(1-\lambda )u-v- \left( \lambda {\theta }_{1}+{\theta }_{1}k \right) s- \left( \lambda {\theta }_{2}+{\theta }_{2}k \right) {\varepsilon }_{t-2}-\cdots - \left( \lambda {\theta }_{q}+{\theta }_{q}k \right) {\varepsilon }_{t-q}}{\alpha (\lambda +k)} + \frac{\mu }{\alpha } } \right\vert $

$=c{ \left\| {L}_{1}-{L}_{2} \right\| }_{\infty }$,

where $c= \left\vert 1-{e}^{ \frac{-b}{\alpha \left( \lambda +k \right) } } \right\vert \sup _{u\in [0,b]} \left\vert {e}^{ \frac{(1-\lambda )u-v- \left( \lambda {\theta }_{1}+{\theta }_{1}k \right) s- \left( \lambda {\theta }_{2}+{\theta }_{2}k \right) {\varepsilon }_{t-2}-\cdots - \left( \lambda {\theta }_{q}+{\theta }_{q}k \right) {\varepsilon }_{t-q}}{\alpha (\lambda +k)} + \frac{\mu }{\alpha } } \right\vert $; 0 ≤ *c* < 1 and *c* is a positive constant.

By Theorem 1, Banach’s Fixed-point Theorem guarantees the existence and uniqueness of the solution for the ARL.

## Additional Information and Declarations

### Data Availability

The following information was supplied regarding data availability: Raw data, including PM 2.5 and CO air pollutant data, and code are available as Supplemental Files.
